# Discoidin domain receptor 1-deletion ameliorates fibrosis and promotes adipose tissue beiging, brown fat activity, and increased metabolic rate in a mouse model of cardiometabolic disease

**DOI:** 10.1016/j.molmet.2020.101006

**Published:** 2020-04-28

**Authors:** Marsel Lino, David Ngai, Alison Liu, Amanda Mohabeer, Cameron Harper, Laura-lee Caruso, Stephanie A. Schroer, Fred Fu, Trevor McKee, Adria Giacca, Minna Woo, Michelle P. Bendeck

**Affiliations:** 1 Department of Laboratory Medicine and Pathobiology, University of Toronto, 1 King's College Circle, Toronto, ON, Canada; 2 Translational Biology and Engineering Program, Ted Rogers Centre for Heart Research, University of Toronto, 661 University Avenue, Toronto, ON, Canada; 3 Toronto General Hospital Research Institute, Department of Medicine, University of Toronto, 101 College Street, Toronto, ON, Canada; 4 STTARR Innovation Center, University Health Network, 101 College Street, Toronto, ON, Canada; 5 Department of Physiology, University of Toronto, 1 King's College Circle, Toronto, ON, Canada

**Keywords:** Obesity, Diabetes, Discoidin domain receptor 1, Collagen, brown fat, Fibrosis

## Abstract

**Objective:**

Discoidin domain receptor 1 (DDR1) is a collagen binding receptor tyrosine kinase implicated in atherosclerosis, fibrosis, and cancer. Our previous research showed that DDR1 could regulate smooth muscle cell trans-differentiation, fibrosis and calcification in the vascular system in cardiometabolic disease. This spectrum of activity led us to question whether DDR1 might also regulate adipose tissue fibrosis and remodeling.

**Methods:**

We have used a diet-induced mouse model of cardiometabolic disease to determine whether DDR1 deletion impacts upon adipose tissue remodeling and metabolic dysfunction. Mice were fed a high fat diet (HFD) for 12 weeks, followed by assessment of glucose and insulin tolerance, respiration via indirect calorimetry, and brown fat activity by FDG-PET.

**Results:**

Feeding HFD induced DDR1 expression in white adipose tissue, which correlated with adipose tissue expansion and fibrosis. Ddr1^−/−^ mice fed an HFD had improved glucose tolerance, reduced body fat, and increased brown fat activity and energy expenditure compared to Ddr1^+/+^ littermate controls. HFD-fed DDR1^−/−^ mice also had reduced fibrosis, smaller adipocytes with multilocular lipid droplets, and increased UCP-1 expression characteristic of beige fat formation in subcutaneous adipose tissue. *In vitro*, studying C3H10T1/2 cells stimulated to differentiate, DDR1 inhibition caused a shift from white to beige adipocyte differentiation, whereas DDR1 expression was increased with TGFβ-mediated pro-fibrotic differentiation.

**Conclusion:**

This study is the first to identify a role for DDR1 as a driver of adipose tissue fibrosis and suppressor of beneficial beige fat formation.

## Highlights

• DDR1 deletion results in decreased obesity, and increased energy expenditure and brown fat activity.
• DDR1 expression was increased in adipose and correlated with obesity and fibrosis.
• DDR1 deletion increased UCP-1 expression in brown and white fat *in vivo*, and in mesenchymal cells *in vitro*.
• *In* *vitro* studies suggest that DDR1 suppresses UCP-1 and drives pro-fibrotic differentiation of mesenchymal cells.

## Graphical abstract

**Figure:**
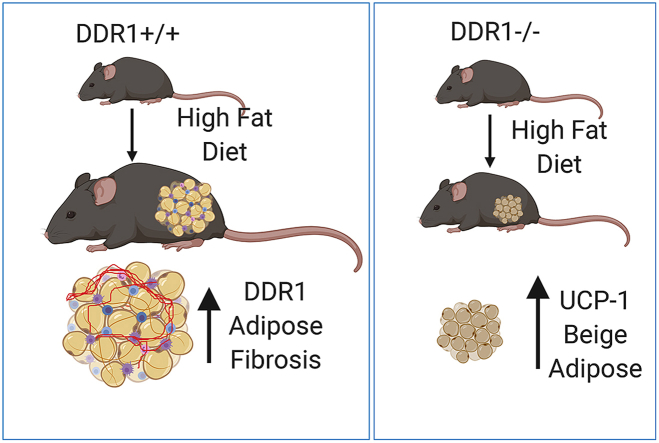

## Abbreviations

α-SMA: α-smooth muscle actin
ABCG1/5: ATP-binding cassette sub-family G member 1/5
ACAT2: acetyl-CoA acetyltransferase 2
BAT: brown adipose tissue
cAMP: cyclic adenosine monophosphate
CD36: cluster of differentiation 36
DDR1: discoidin domain receptor 1
eFat: epididymal fat
FAS: Fatty acid synthase
FDG-PET: ^18^fluorodeoxyglucose - positron emission tomography
GTFRIRD1: general transcription factor II-I repeat domain-containing protein 1
HFD: high-fat diet
HSL: hormone sensitive lipase
LPL: lipoprotein lipase
MetS: metabolic syndrome
MRTF-A: myocardin-related transcription factor-A
PKA: protein kinase A
PPARγ: peroxisome proliferator activated receptor γ
sFat: subcutaneous fat
SREBP2: sterol regulatory element-binding protein 2
UCP-1: uncoupling protein-1

## 1 Introduction

Type 2 diabetes (T2D) and metabolic syndrome (MetS) affect millions of people worldwide and have reached epidemic proportions [1]. The disorders underlying these systemic syndromes include insulin resistance, hypertriglyceridemia, hepatic steatosis and obesity [2]. Resulting from caloric excess, obesity is characterized by the rapid expansion of adipose tissue, which requires extensive remodeling of the extracellular milieu, the maturation of adipose progenitors, and adequate neovascularization to provide expanding adipose tissue with requisite nutrients and oxygen [3,4]. Excessive adipose tissue expansion can be accompanied by hypoxia, inflammation, and fibrosis, which lead to adipose tissue dysfunction, an early step in the progression towards insulin resistance and T2D [4]. Thus the development of therapeutic and lifestyle interventions aimed at reducing obesity are instrumental in curbing the current global metabolic syndrome and diabetes epidemic.

Brown adipose tissue (BAT) is a specialized type of fat found in discrete depots, including the interscapular region, and is characterized by high levels of uncoupling protein-1 (UCP-1), which uncouples cellular respiration, resulting in increased energy expenditure in the form of thermal energy [5]. Brown adipose tissue can also be found within white adipose tissue and here it is referred to as beige fat. Beige fat can be induced by cold-exposure, which activates the β3-adrenergic receptor and signals via cyclic adenosine monophosphate (cAMP)/protein kinase A (PKA) to activate PR domain-containing protein 16 (PRDM16), the key transcriptional regulator that drives brown and beige adipogenesis and UCP-1 expression [6]. The consequent increase in energy expenditure can lead to reductions in obesity [7]. The discovery that cold-inducible, metabolically active brown adipose tissue exists in adult humans [8] has resulted in a growing interest in developing pharmaceutical agents that induce beige fat to combat obesity-related disorders, including T2D [7].

Recent studies have demonstrated a reciprocal relationship between adipose tissue fibrosis and beiging. In a mouse model of obesity, the deletion of fibrotic type VI collagen resulted in an improved metabolic phenotype, including reduced body weight, increased energy expenditure, and improved lipid clearance and glucose tolerance [9]. Myocardin-related transcription factor-A (MRTF-A) drives fibrosis by inducing pre-adipocyte differentiation into fibrotic cells [10]. The deletion of MRTF-A in high-fat fed mice improved metabolic parameters by increasing beige fat [11]. These studies demonstrate that fibrosis can suppress beige fat formation. Conversely, beige adipogenesis can suppress fibrosis. General transcription factor II-I repeat domain-containing protein 1 (GTF2IRD1) was recently identified as a cold-inducible transcription factor that complexes with PRDM16 to repress fibrosis-related gene expression [12]. Understanding the interplay between adipose tissue fibrosis and beiging can further our understanding of adipose tissue dysfunction.

Discoidin domain receptor 1 (DDR1) is a receptor tyrosine kinase that binds triple helical collagens via its extracellular discoidin domain [13,14] and functions in extracellular matrix remodeling and fibrosis [15,16] as well as cell differentiation and migration [[17], [18], [19]]. Previous work from our laboratory established an important role for DDR1 in atherosclerosis [[20], [21], [22]]. Recently, we studied the role of DDR1 in a mouse model of cardiometabolic disease, using Ldlr^−/-^ mice fed a high fat diabetogenic diet (HFD), and discovered that DDR1 promotes vascular calcification, promoting the trans-differentiation of vascular smooth muscle cells to osteochondrocytic cells. DDR1 stimulated PI3K/Akt signaling, activating the master osteogenic regulator Runt-related transcription factor 2 (RUNX2) [23]. This was interesting because the differentiation of mesenchymal stem cells into osteocytes or adipocytes is driven by RUNX2 or PPARγ, respectively [24]. The fact that DDR1 could regulate RUNX2, cell trans-differentiation and fibrosis in the vascular system led us to question whether DDR1 might also regulate adipose tissue differentiation and remodeling in obesity. We have used a diet-induced mouse model of atherosclerosis, metabolic disease and obesity to determine whether DDR1 deletion impacts upon adipose tissue remodeling and metabolic dysfunction.

## 2 Materials and methods

### 2.1 Animals

Animal experiments were performed in accordance with the guidelines of the Canada Council on Animal Care, with the approval of the University of Toronto, Faculty of Medicine Animal Care Committee. Ddr1^+/+^; Ldlr^−/-^ (Ddr1^+/+^) and Ddr1^−/−^; Ldlr^−/-^ (Ddr1^−/−^) on a mixed background (129/Sv x C57Bl/6N), and Ddr1^+/+^ (Ddr1^+/+(BL6)^), and Ddr1^−/−^ (Ddr1^−/−(BL6)^) mice on the C57Bl/6N background were generated as described [22]. Mice were generated by breeding female Ddr1+/− heterozygotes with male Ddr1−/− mice because female Ddr1−/− mice do not breed well. At 6 weeks of age, male mice were placed on a Western-type high-fat diet (40% fat, 43% carbohydrate, 0.5% cholesterol; D05011404, Research Diets, New Brunswick, NJ) for 12 weeks to induce obesity and glucose intolerance. Animals were fasted over-night (16 h) prior to assessment of plasma parameters. Plasma samples were collected using K2 EDTA-coated Microvette® capillary tubes (16.444.100; Sarstedt, Numbrecht, DE). Plasma was separated by centrifugation at 5,000 rpm for 5 min. Plasma parameters were assayed using the Beckman AU480 Biochemistry Analyzer at the Toronto Centre for Phenogenomics. Blood glucose was measured by tail-vein bleed using the OneTouch® Ultra® 2 glucometer. Oral glucose tolerance tests (GTTs) were performed by administering 2 g/kg *d*-glucose after an over-night fast. Insulin tolerance tests (ITTs) were performed after a 5-hour fast, followed by intra-peritoneal administration of insulin (0.75 U/kg). Animals were euthanized and tissues were isolated for analysis. Liver, epididymal adipose, inguinal adipose, brown adipose, and muscle, were isolated and fixed in 4% paraformaldehyde for 24 h to prepare for immunohistochemical analysis, or snap-frozen for protein analysis by immunoblot.

### 2.2 Histology and immunohistochemistry

To visualize hepatic lipid accumulation, liver sections from Ddr1^+/+^ and Ddr1^−/−^ mice fed HFD were stained with Oil Red O (ORO) and H&E. Pancreatic islets of Langerhans were visualized by immunostaining for insulin. Staining of the liver and pancreas was performed by the Toronto Centre for Phenogenomics. For adipocyte size measurements, adipose tissue sections were stained for H&E. A minimum of 12 fields (20X magnification) were captured per tissue. Images were converted to black and white, and linear contrast enhancement was applied to improve the visualization of cell borders. This enabled the detection of cells by applying a threshold on the white channel. Cell size frequency was determined by binning individual cell sizes into discrete size ranges and determining the frequency of cells in each range. Additionally, the cell area was calculated and expressed as mean adipocyte size. For adipose fibrosis measurements, a minimum of five fields (10X magnification) were captured per tissue. Peri-adipocyte PSR stain was detected by drawing a region of interest (ROI) around a population of adipocytes and applying a threshold for red. The PSR-stained area was quantified and expressed as a percentage of PSR-stained area relative to ROI area. Adipose tissue sections were stained with UCP-1 antibody (ab10983; Abcam, Cambridge, UK) and HRP-linked rabbit secondary (7074; Cell Signaling Technology, Danvers, MA). Staining was visualized using a 3,3′-diaminobenzidine (DAB) kit (8059; Cell Signaling Technology). Images were captured using the Nikon Eclipse Ci microscope and analyzed using NIS Elements Software (Nikon, Tokyo, JP).

### 2.3 Assessment of lean/fat mass by computed tomography (CT)

Lean/fat measurement was performed on CT images of Ddr1^+/+^ and Ddr1^−/−^ mice fed HFD for 12 weeks. Mice were first manually contoured using the software ITK-SNAP 3.8.0 as described [25] in order to remove the bed. Subsequent processing was automated using the Insight Toolkit (ITK) 5.0.0 package in Python using empirically determined parameters and following a procedure similar to that described in [26]. First, CT intensities were normalized to the Hounsfield scale. A Gaussian blur was applied to the image with σ = 0.1 mm to smooth out noise and background voxels were removed by a binary threshold applied at −700 HU. The largest connected object was taken to be the mouse body. Next, to measure fat inside the segmented body, “candidate” fat voxels were first segmented by applying a threshold between −250 and 50 HU. Then, to correct for partial volume effect occurring at the air/tissue interface, which could result in an overestimated fat measurement, additional voxels were excluded. This was done by finding regions of air (below −250 HU), removing any small speckles in the process, and applying a binary dilation filter to grow these areas by 3 pixels. For the lung region (the largest region of air inside the mouse body), an additional 3-pixel dilation was applied. These dilated regions were then used to mask out the candidate fat voxels. Finally, of the remaining fat voxels, any small connected objects (speckles or holes) were removed, resulting in the final fat segmentation. Percentage fat was reported using whole body volume, excluding air, as the denominator [25,26].

### 2.4 Assessment of Whole-body metabolic activity by indirect calorimetry

Analysis of metabolic parameters was performed *in vivo* as previously described using the Comprehensive Laboratory Animal Monitoring System (CLAMS; Columbus Instruments, Columbus, OH) [27]. Energy expenditure, food intake, oxygen consumption (VO_2_), carbon dioxide production (VCO_2_), respiratory exchange ratio (RER), and locomotor activity were assessed in Ddr1^+/+^ and Ddr1^−/−^ mice fed an HFD for 6 weeks (6wk HFD). Mice were acclimatized in the metabolic chambers for 24 h prior to the start of data collection, followed by a 24-hour period of data collection. Data was categorized as diurnal (light cycle) and nocturnal (dark cycle). Data was analyzed using CLAX Software (Columbus Instruments).

### 2.5 Assessment of cold-induced brown fat activity using ^18^fluorodeoxyglucose-positron emission tomography (FDG-PET) and scintillation counts

BAT activity was assessed in Ddr1^+/+^ and Ddr1^−/−^ mice fed an HFD for 12 weeks. Briefly, to induce BAT activation, mice were exposed to cold (4 °C) for 4 h prior to FDG-PET. ^18^FDG was administered by intra-peritoneal injection 1 h prior to scan to allow for uptake. Micro-CT and micro-PET images were acquired on GE Locus micro-CT and Siemens Inveon micro-PET (Siemens Healthcare Molecular Imaging, Knoxville, TN) systems, respectively, and were imported into the Siemens Inveon Research Workstation 4.0 software (Siemens Healthcare Molecular Imaging) for quantitative assessment of ^18^FDG uptake in BAT. PET and CT images were aligned using semi-automated rigid body registration with manual fine tuning. Regions of interest containing the full extent of the brown fat pad were identified manually, using the micro-CT primarily as a guide, identifying regions of low HU intensity corresponding to fat and avoiding muscle and bone. A series of axial regions of interest were contoured by hand, spaced every 3–4 CT slices apart, and the full volume was then generated by interpolating between the axial regions of interest to produce a 3D volume corresponding to the BAT. ^18^FDG uptake within BAT was quantified and expressed as a mean intensity in units of percent injected dose per gram (%ID/g). To verify the accuracy of the FDG-PET method, BAT was excised from mice immediately after FDG-PET, along with eFat, sFat, and muscle tissue. Radioactivity (γ-count) in excised tissue was measured by scintillation counter and expressed as %ID/g, normalized to tissue weight. Then, %ID/g values obtained by FDG-PET image analysis were correlated to %ID/g values determined by scintillation counts.

### 2.6 Immunoblot

Tissues were snap-frozen and ground using a mortar and pestle. Protein was isolated from tissue and cell lysates using 1x Cell Lysis Buffer (9803; Cell Signaling Technology). Antibodies were obtained from Cell Signaling Technology unless otherwise specified: DDR1 (5583); UCP-1 (ab10983; Abcam); phospho-HSL (4139); HSL (4107); FAS (3180); Perilipin (9349); PPARγ (2435); phospho-PKA substrate (9624S); MRTF-A (14760); collagen-1 (ab21286; Abcam); α-smooth muscle actin (14968); histone H3 (ab1791; Abcam); β-actin (4967); HRP-linked rabbit secondary (7074); HRP-linked mouse secondary (7076). Immunoblots were imaged using the ChemiDoc™ MP imaging system and quantified using the Image Lab™ Software (Bio-Rad Laboratories).

### 2.7 mRNA expression analyses

Total RNA was isolated from sFat tissue using the RNeasy Lipid Tissue Mini Kit (74084; QIAGEN, Hilden, DE). Briefly, sFat tissues were snap-frozen in liquid nitrogen and homogenized using a mortar and pestle over dry ice. Concentration and RNA purity were determined using a NanoDrop 1000 spectrophotometer (Thermo Fischer Scientific, Waltham, MA). RNA samples were treated with DNase I (18068015; Life Technologies, Carlsbad, CA) and reverse-transcribed into cDNA using the SuperScript First-Strand Synthesis Kit (11904018; Life Technologies) per the manufacturer's instructions. cDNA was diluted 2-fold (4-fold for *18s*) prior to mixing with Power SYBR Green PCR Master Mix (4367659; Life Technologies) and the appropriate primers for real time RT-PCR amplification. Primer sequences are listed in Supplemental Table S1. Primers for genes involved in lipid metabolism were previously published [28]. All other primers were designed using Batch Primer 3 v1.0. Data was analyzed using Bio-Rad CFX Manager Software 3.0 (Bio-Rad). Target gene expression was normalized to *18s* and expressed as a fold change relative to wild-type control (Ddr1^+/+^) samples via the 2^- Δ*C*^_T_ method [29].

### 2.8 Cell culture

C3H10T1/2 mesenchymal stem cells (a gift from Dr. Paul Hamel; University of Toronto) were used between passages 5–10. Cells were propagated in DMEM supplemented with 10% FBS and 1% penicillin-streptomycin (Thermo Fischer Scientific). Cell culture media and reagents were obtained from Thermo Fischer Scientific unless otherwise specified. Plasmid containing full-length DDR1b isoform (a gift from the late Dr. Wolfgang Vogel) was transfected into C3H10T1/2 cells using Lipofectamine-3000 (L3000) according to the manufacturer's instructions. Adipogenic and pro-fibrotic differentiation of C3H10T1/2 cells was performed as previously described [11,30,31]. Pre-confluent C3H10T1/2 cells were treated with 50 ng/mL BMP-4 (PHC9534) to induce differentiation into white adipocytes, with 6.3 nM BMP-7 (PHC9544) to induce brown adipocyte differentiation or with 1 nM TGFβ (PHG9214) to induce pro-fibrotic differentiation. Once confluent, cells were cultured in media supplemented with 5 μM dexamethasone (D1756; Sigma-Aldrich), 0.5 mM 3-isobutyl-1-methylxanthine (IBMX) (I5879; Sigma-Aldrich), 860 nM insulin (I0908; Sigma-Aldrich), 1 nM 3,3,5-triiodo-l-thyronine (T3) (T6397; Sigma-Aldrich), and 125 μM indomethacin (I8280; Sigma-Aldrich) for 2 days, and maintained for an additional 6 days in media supplemented with 860 nM insulin and 1 nM T3. DDR1 inhibition was achieved using 1 mM DDR1IN1 (5077; Tocris Bioscience, Bristol, UK). Adipogenesis was visualized by Oil Red-O stain (O0625; Sigma-Aldrich).

### 2.9 Statistical analyses

Data were analyzed using GraphPad Prism Software (La Jolla, California, USA). Normality was determined by D'Agostino-Pearson omnibus test. Parametric and non-parametric analyses were performed as indicated in the Figure legends. Data is presented as mean ± SEM.

## 3 Results

### 3.1 DDR1 deficient mice have reduced body Weight and adiposity after feeding HFD for 12 Weeks

**Figure 1:**
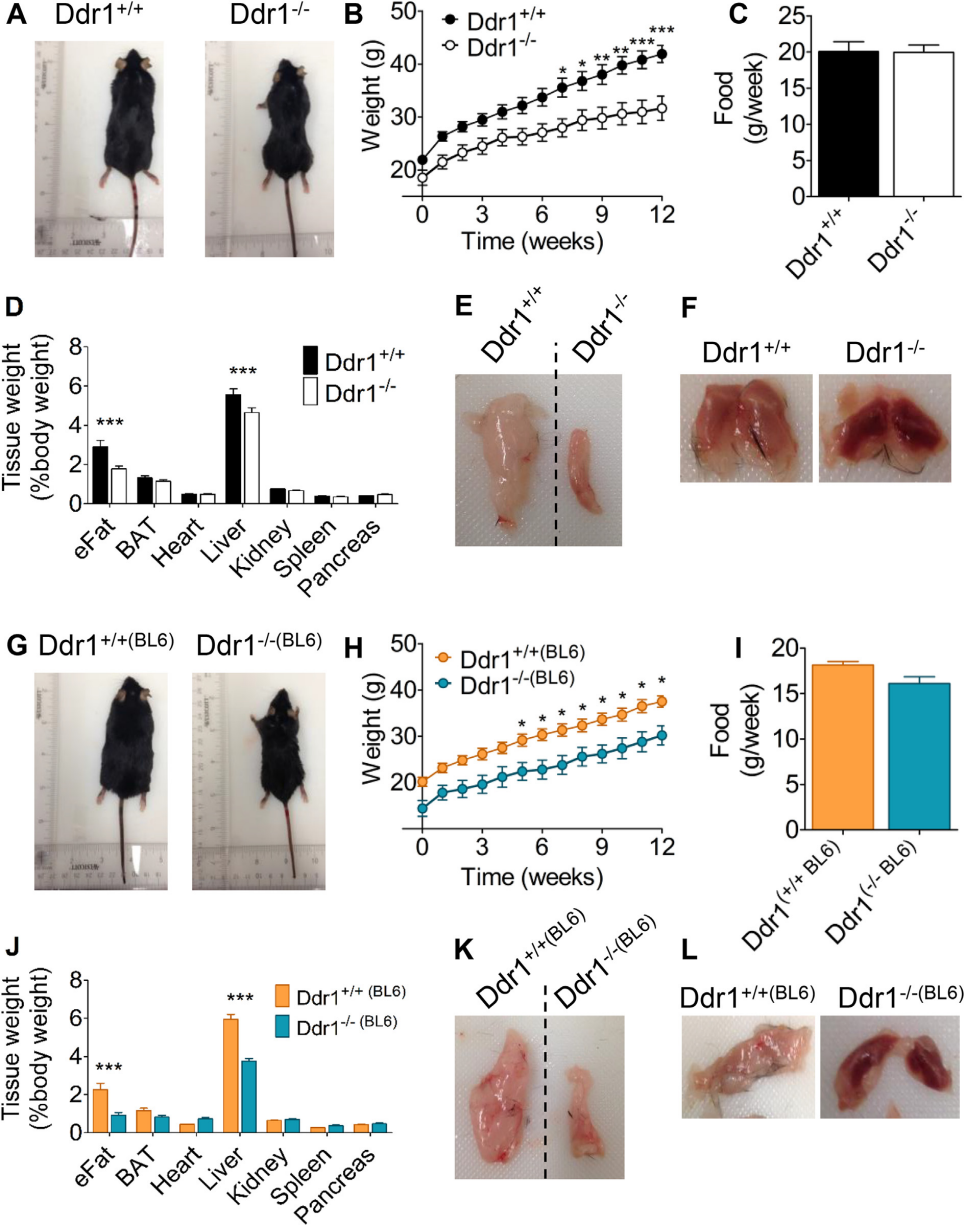
**DDR1 deficient mice have reduced body weight and adiposity after feeding HFD for 12 weeks.** (**A-B**) Ddr1^+/+^; Ldlr^−/-^ (Ddr1^+/+^; n = 13) and Ddr1^−/−^; Ldlr^−/-^ (Ddr1^−/−^; n = 13) mice (**A**) were fed high-fat diet (HFD) for 12 weeks and weighed weekly (**B**). (**C**) Food consumption was recorded. (**D**) Tissue weights were recorded after HFD. (**E-F**) Epididymal (**E**) and interscapular (**F**) adipose tissue from Ddr1^+/+^ and Ddr1^−/−^ mice fed HFD. (**G-I**) Ddr1^+/+^ (Ddr1^+/+(BL6)^; n = 11) and Ddr1^−/−^ (Ddr1^−/−(BL6)^; n = 12) mice (**G**) on C57BL6 background were fed high-fat diet (HFD) for 12 weeks and body weight (**H**) and food consumption (**I**) were recorded. (**J**) Tissue weights were recorded. (**K-L**) Epididymal (**K**) and interscapular (**L**) adipose tissue from Ddr1^+/+(BL6)^ and Ddr1^−/−(BL6)^ mice fed HFD. Statistical analysis was performed by 2-way ANOVA with Bonferroni post-hoc test (**B**, **D**, **H**, and **J**), and student's t-test (**C** and **I**). The values are mean ± SEM. ∗p < 0.05, ∗∗p < 0.01, ∗∗∗p < 0.001.

We used a mouse model of cardiometabolic disease, feeding an HFD to Ldlr^−/-^ mice [32]. We studied Ddr1^+/+^; Ldlr^−/-^ (Ddr1^+/+^) and Ddr1^−/−^; Ldlr^−/-^ (Ddr1^−/−^) mice. To eliminate possible confounding effects of the Ldlr-deletion, we also report results from mice on C57BL/6N background (Ddr1^+/+(BL6)^ and Ddr1^−/−(BL6)^) fed the HFD for 12 weeks. Ddr1^−/−^ mice were smaller after 12 weeks on the HFD (Figure 1A). There was no significant difference in weight between genotypes from 0 to 6 weeks on HFD; however, after 6 weeks through to 12 weeks of HFD, Ddr1^−/−^ mice weighed significantly less compared to Ddr1^+/+^ mice (Figure 1B), despite no difference in food consumption (Figure 1C). Epididymal adipose tissue (eFat) was significantly smaller in Ddr1-deficient mice fed an HFD (Figure 1D,E), and inter-scapular adipose tissue (BAT) had a darker appearance (Figure 1F) compared to littermate controls. The Ddr1^−/−^ mice had significantly reduced total plasma cholesterol due to a reduction in LDL-C (Supplemental Table S2; data previously published in [23]). There was no significant difference in fasting plasma triglycerides and fasting blood glucose between Ddr1^+/+^ and Ddr1^−/−^ mice fed an HFD (Supplemental Table S2). The findings were similar in C57BL/6N mice. Ddr1^+/+(BL6)^ and Ddr1^−/−(BL6)^ mice were fed an HFD for 12 weeks and the Ddr1^−/−(BL6)^ mice were smaller (Figure 1G). Body weight was significantly reduced in Ddr1^−/−(BL6)^ mice from weeks 5 through 12 on an HFD (Figure 1H), whereas food consumption did not differ between Ddr1^+/+(BL6)^ and Ddr1^−/−(BL6)^ mice (Figure 1I). Ddr1^−/−(BL6)^ mice also had significantly smaller eFat (Figure 1J,K), as well as darker BAT (Figure 1L). These findings show that Ldlr deficiency does not confound the DDR1 deficient phenotype. Taken together, these findings reveal that Ddr1 deficiency correlates with reduced obesity.

### 3.2 DDR1 deficient mice have improved metabolic function after 12 Weeks on HFD

**Figure 2:**
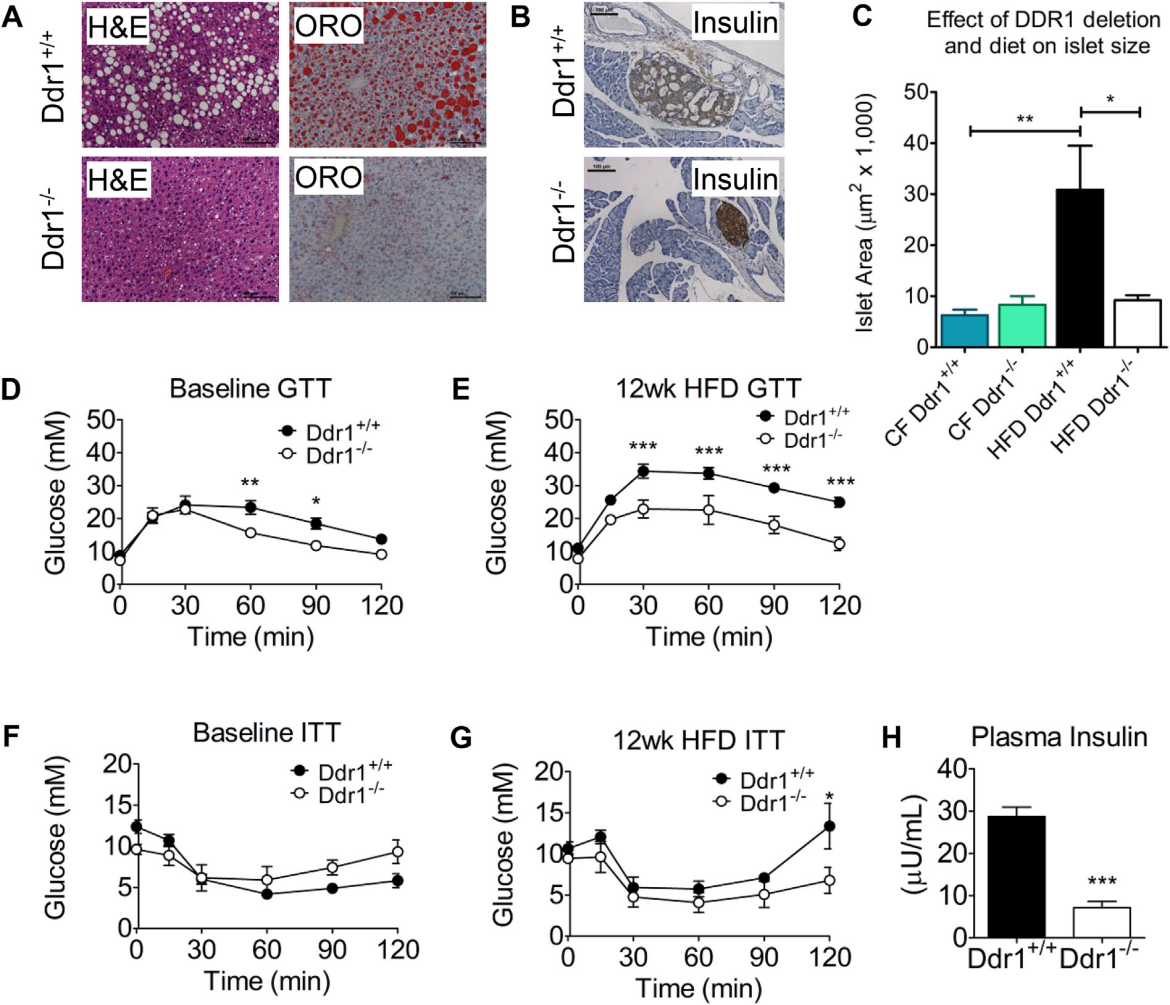
**DDR1 deficient mice have improved metabolic function after 12 weeks on HFD.** (**A**) Liver hematoxylin and eosin (H&E) and Oil Red O (ORO) staining obtained from Ddr1^+/+^ and Ddr1^−/−^ mice fed HFD. (**B**) Insulin staining on pancreatic sections obtained from Ddr1^+/+^ and Ddr1^−/−^ mice. (**C**) Pancreatic islet area was determined in Ddr1^+/+^ and Ddr1^−/−^ mice fed normal chow diet (CF) or HFD for 12 weeks (n = 6). (**D-E**) Oral glucose tolerance test (GTT) was performed in Ddr1^+/+^ and Ddr1^−/−^ mice before (**D**; n = 8) and after 12 weeks on HFD (**E**; n = 8). (**F-G**) Insulin tolerance test (ITT) was performed in Ddr1^+/+^ and Ddr1^−/−^ mice before (**F**; n = 7) and after 12 weeks on HFD (**G**; n = 7). (**H**) Fasting circulating insulin levels were determined in Ddr1^+/+^ and Ddr1^−/−^ mice fed HFD (n = 6). Statistical analysis was performed by 2-way ANOVA with Bonferroni post-hoc test (**D**, **E**, **F**, and **G**), and student's t-test (**C** and **H**). The values are mean ± SEM. ∗p < 0.05, ∗∗p < 0.01, ∗∗∗p < 0.001.

Next, we assessed metabolic parameters in HFD-fed Ddr1^+/+^ and Ddr1^−/−^ mice. Consistent with reduced body weight and adiposity, hepatic steatosis was evident in Ddr1^+/+^ mice fed an HFD, while minimal lipid was detected in Ddr1^−/−^ mice (Figure 2A). This was associated with a significant reduction in liver weight in Ddr1^−/−^ compared to Ddr1^+/+^ mice (Figure 1D). Pancreatic sections were stained for insulin to visualize the islets of Langerhans (Figure 2B). Ddr1^+/+^ and Ddr1^−/−^ islet size was not different in age-matched control mice fed regular chow for 12 weeks (Figure 2C), thus we conclude that normal islet size was not affected by DDR1 deletion. Instead, an increase in islet size occurred in response to the high fat diet challenge in Ddr1^+/+^ mice but not Ddr1^−/−^ mice after 12 weeks on an HFD (Figure 2C). DDR1-deficient mice had improved baseline glucose tolerance even before the HFD (Figure 2D) and much improved glucose tolerance after 12 weeks on an HFD (Figure 2E). There were no significant differences in baseline glucose during insulin tolerance test (Figure 2F), but after 12 weeks on an HFD, 2-hour glucose levels were reduced significantly during the insulin tolerance test (Figure 2G). Consistent with this, Ddr1^+/+^ mice on an HFD had elevated plasma insulin compared to Ddr1^−/−^ mice, which had normal insulin levels (Figure 2H). These findings demonstrate that the loss of DDR1 ameliorates the development of hepatic steatosis and pancreatic defects associated with the metabolic syndrome phenotype.

### 3.3 DDR1 protein expression is induced in White adipose tissue and liver after 12 Weeks on HFD

**Figure 3:**
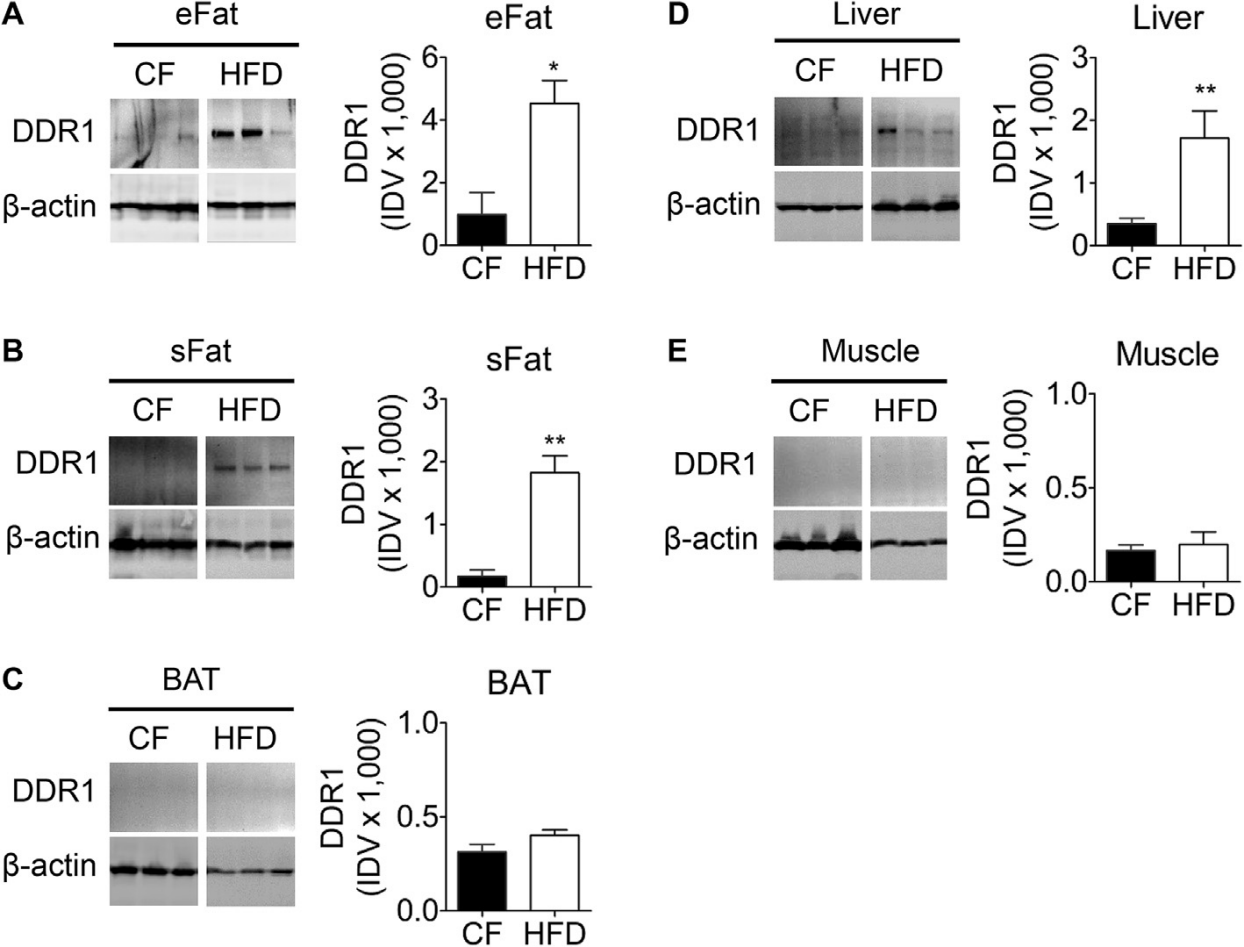
**DDR1 protein expression is induced in white adipose tissue and liver after 12 weeks on HFD.** (**A-E**) DDR1 expression was assessed by immunoblot in epididymal adipose (**A**; eFat), subcutaneous adipose (**B**; sFat), interscapular brown adipose (**C**; BAT), liver (**D**), and muscle (**E**) tissues isolated from Ddr1^+/+^ mice after 12 weeks on CF or HFD (n = 6). Immunoblots were quantified and normalized to β-actin and expressed as integrated density values (IDV). Statistical analysis was performed by two-tailed student's t-test. The values are mean ± SEM. ∗p < 0.05, ∗∗p0.01.

To investigate the role of DDR1 in metabolic dysfunction, we probed peripheral adipose, liver, and muscle for DDR1 expression in mice fed normal chow diet (CF) or an HFD (Figure 3). DDR1 expression was significantly increased in response to HFD-feeding in eFat (Figure 3A), subcutaneous fat (sFat; Figure 3B), and liver (Figure 3D). By contrast, DDR1 expression was negligible in BAT (Figure 3C) and muscle (Figure 3E). These findings suggest that DDR1 expression is correlated with HFD-induced peripheral adipose tissue expansion and remodeling, and hepatic steatosis.

### 3.4 DDR1 deficient mice have reduced adipocyte size in epididymal and subcutaneous adipose tissue after 12 Weeks on HFD

**Figure 4:**
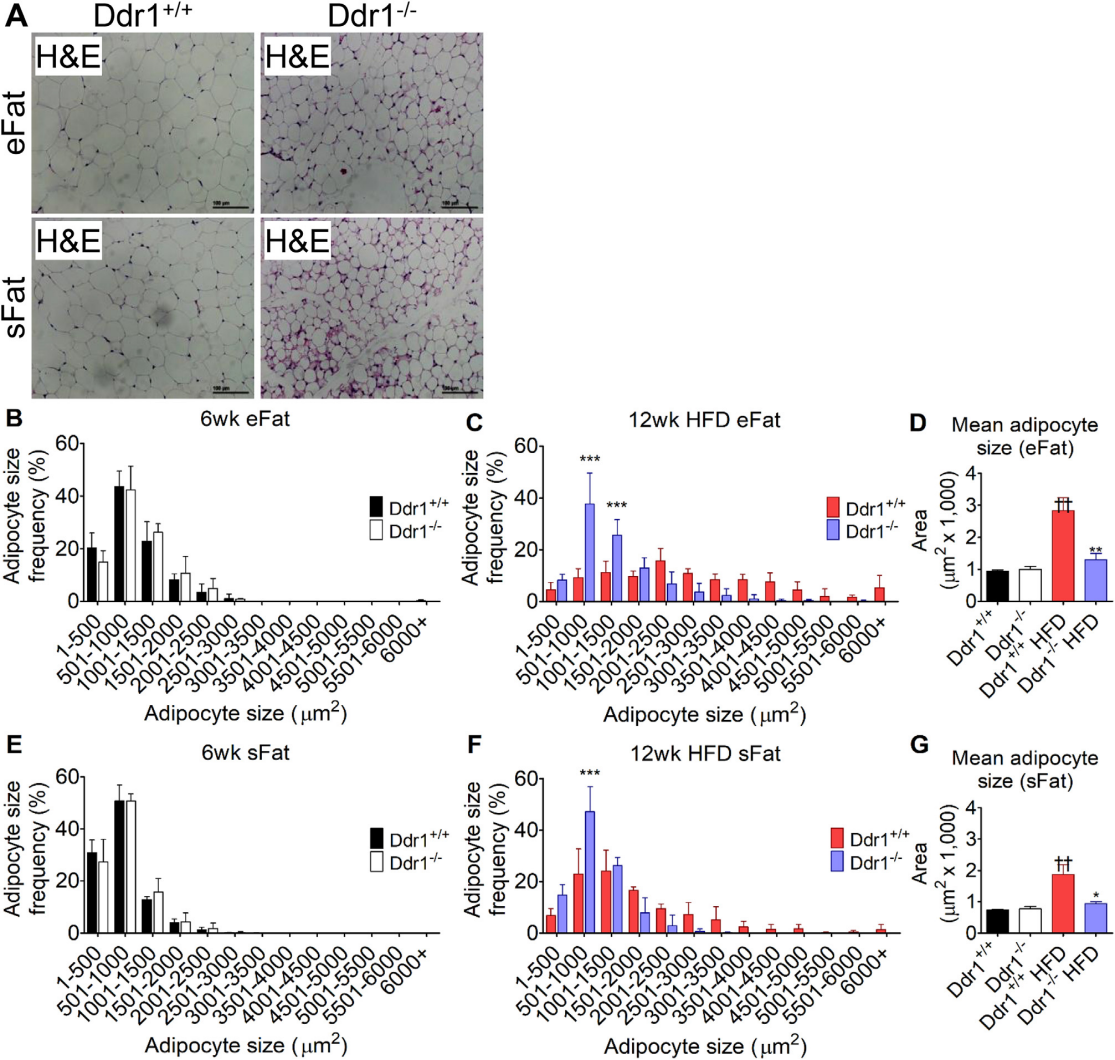
**DDR1 deficient mice have reduced adipocyte size after 12 weeks on HFD.** (**A**) Epididymal (eFat) and subcutaneous (sFat) tissues were isolated from Ddr1^+/+^ and Ddr1^−/−^ mice and stained with H&E to visualize adipocyte size (scale bars = 100 μm). (**B–C**) Adipocyte size distribution was measured from H&E stains of eFat before (**B**; 6wk) and after (**C**; 12wk) on HFD (n = 3). (**D**) Mean adipocyte size was determined in eFat before and after HFD-feeding (n = 3). (**E-F**) Adipocyte size distribution in sFat before (**E**; 6wk) and after (**F**; 12wk) HFD-feeding. (**G**) Mean adipocyte size in sFat before and after HFD (n = 3). Statistical analysis was performed by 2-way ANOVA with Bonferroni post-hoc test (**B**, **C**, **E**, and **F**) and by 1-way ANOVA with Bonferroni post-hoc test (**D** and **G**). The values are mean ± SEM. ∗p < 0.05, ∗∗p < 0.01, ∗∗∗p < 0.001, comparing Ddr1^+/+^ to Ddr1^−/−^. ††p < 0.01, comparing HFD-fed to pre-HFD-feeding.

Sections from eFat and sFat were stained with H&E after 12 weeks on an HFD (Figure 4A). DDR1 deficient mice had smaller adipocytes compared to littermate controls, and multi-locular lipid droplets were observed in sFat from Ddr1^−/−^ but not Ddr1^+/+^ mice fed an HFD (Figure 4A). To assess changes in adipocyte size, measurements were taken from mice at 6 weeks of age (age matched baseline from CF mice) and compared to mice fed an HFD for 12 weeks. In eFat at baseline, adipocyte size distribution was similar in Ddr1^+/+^ and Ddr1^−/−^ mice (Figure 4B). After 12 weeks on the HFD, the adipocyte size distribution in Ddr1^+/+^ mice was shifted to the right, indicating an increase in the frequency of larger adipocytes, while in Ddr1^−/−^ mice, adipocyte size distribution was unchanged from baseline (Figure 4C). Mean adipocyte size in eFat was similar in both mouse genotypes at baseline whereas after 12 weeks on an HFD, there was a nearly 3-fold increase in mean adipocyte size in Ddr1^+/+^ mice, while adipocyte size was significantly smaller in Ddr1^−/−^ mice and not significantly different than baseline (Figure 4D). Similar observations were made in sFat (Figure 4E–G). At baseline, adipocyte size distribution was similar in Ddr1^+/+^ and Ddr1^−/−^ mice (Figure 4E), but after 12 weeks on an HFD, adipocyte size distribution shifted to the right in Ddr1^+/+^ mice but not in Ddr1^−/−^ mice (Figure 4F). Mean adipocyte size in sFat was significantly higher in Ddr1^+/+^ mice after 12 weeks on an HFD, while the mean adipocyte size was significantly smaller in Ddr1^−/−^ mice and not significantly different than baseline (Figure 4G). Taken together, these findings demonstrated that DDR1 deficiency prevented the HFD-induced increase in adipocyte size in epididymal and subcutaneous adipose tissue and was associated with the appearance of adipocytes with multilocular lipid droplets within subcutaneous adipose tissue of Ddr1^−/−^ mice.

### 3.5 DDR1 deficient mice have reduced body fat, increased energy expenditure and increased cold-induced BAT activity

**Figure 5:**
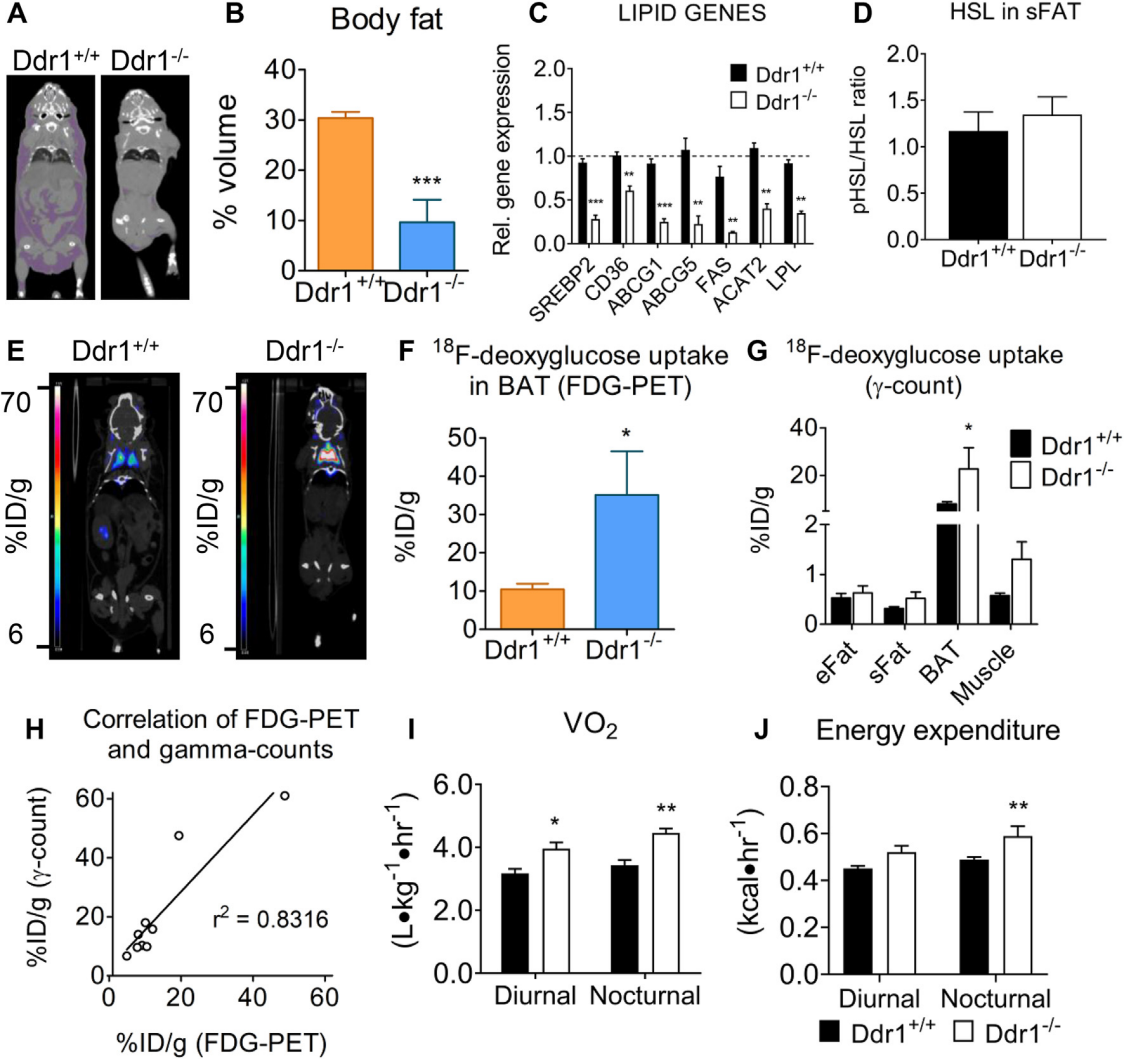
**DDR1 deficient mice have reduced body fat, increased energy expenditure and increased cold-induced BAT activity.** (**A-B**) Body fat was determined by analyzing CT images of Ddr1^+/+^ and Ddr1^−/−^ mice fed HFD (**A**) and expressed as a percentage of total body volume (**B**) (Ddr1^+/+^ n = 5; Ddr1^−/−^ n = 4). (**C**) Expression of genes involved in lipid transport (CD36, ABCG1/5), lipid synthesis (SREBP2, FAS, ACAT2), and lipolysis (LPL) were measured. (**D**) Active (phosphorylated) HSL was measured in sFat from Ddr1^+/+^ and Ddr1^−/−^ mice fed HFD (Ddr1^+/+^ n = 6; Ddr1^−/−^ n = 5). (**E**) Ddr1^+/+^ and Ddr1^−/−^ mice were fed HFD for 12 weeks and exposed to cold (4 °C) for 4 h prior to assessing BAT activity by FDG-PET (Ddr1^+/+^ n = 5; Ddr1^−/−^ n = 4). (**F**–**G**) BAT activity was assessed by volumetric analysis of PET scans (**F**) as well as by scintillation counting on excised tissue (**G**), to determine the percentage of injected ^18^F-deoxyglucose dose/gram tissue (%ID/g) within BAT (Ddr1^+/+^ n = 5; Ddr1^−/−^ n = 4). (**H**) Correlation of the results obtained from volumetric analysis of PET scans and γ-count. (**I**–**J**) Ddr1^+/+^ and Ddr1^−/−^ mice were fed HFD for 6 weeks and placed in metabolic chambers for 48 h to assess metabolic activity by measuring VO_2_ (**I**; n = 6) and energy expenditure (**J**; n = 6). Statistical analysis was performed by student's t-test (**B**, **C**, **D**, and **F**), 2-way ANOVA with Bonferroni post-hoc test (**G**, **I**, and **J**), and linear regression analysis (**H**). The values are mean ± SEM. ∗p < 0.05, ∗∗p < 0.01 compared to Ddr1^+/+^.

Body fat was measured from computerized tomography images of mice (Figure 5A). There was a 60% reduction in body fat in Ddr1^−/−^ compared to Ddr1^+/+^ mice after 12 weeks of an HFD (Figure 5B). Next, we measured the expression of mRNA for genes involved in lipid transport (*cd36*, *abcg1*, *abcg5*), lipid synthesis (*srebp2*, *fas*, *acat2*), and lipolysis (*lpl*) in sFat from 12-week HFD-fed mice and found all were significantly decreased in Ddr1^−/−^ mice (Figure 5C). Lipolysis was also measured by immunoblotting for total and phosphorylated hormone-sensitive lipase (HSL) in sFat of HFD-fed mice and there was no difference between Ddr1^+/+^ and Ddr1^−/−^ mice (Figure 5D). To assess metabolic activity in the brown adipose tissue (BAT), mice were fasted overnight and exposed to cold (4 °C) for 4 h followed by measurement of ^18^F-deoxyglucose uptake using positron emission scanning (Figure 5E). BAT metabolic activity, as assessed on volumetric PET scans (Figure 5F), and scintillation counting on excised tissue (Figure 5G), was significantly increased in Ddr1^−/−^ compared to Ddr1^+/+^ mice. There was significant correlation between the PET and scintillation count measures (Figure 5H). To assess whole body metabolism, we used indirect calorimetry to measure energy expenditure in Ddr1^+/+^ and Ddr1^−/−^ mice fed HFD for 6 weeks. Diurnal and nocturnal oxygen consumption (VO_2_) was significantly higher in Ddr1^−/−^ mice compared to Ddr1^+/+^ mice (Figure 5I). Ddr1^−/−^ mice also had significantly elevated nocturnal energy expenditure (Figure 5J). VCO_2_ was also increased in Ddr1^−/−^ mice, whereas respiratory exchange ratio (RER) and locomotor activity were unchanged (Supplemental Fig. S1). These data demonstrate that Ddr1^−/−^ mice have increased energy expenditure without changes in locomotor activity. Taken together, these results demonstrate that Ddr1^−/−^ mice fed an HFD have decreased body fat, and increased BAT activity and whole-body metabolic rate.

### 3.6 DDR1 deficient mice have increased UCP-1 mRNA and protein expression in sFat and BAT, while DDR1 overexpression suppresses UCP-1 during adipogenic differentiation *in vitro*

**Figure 6:**
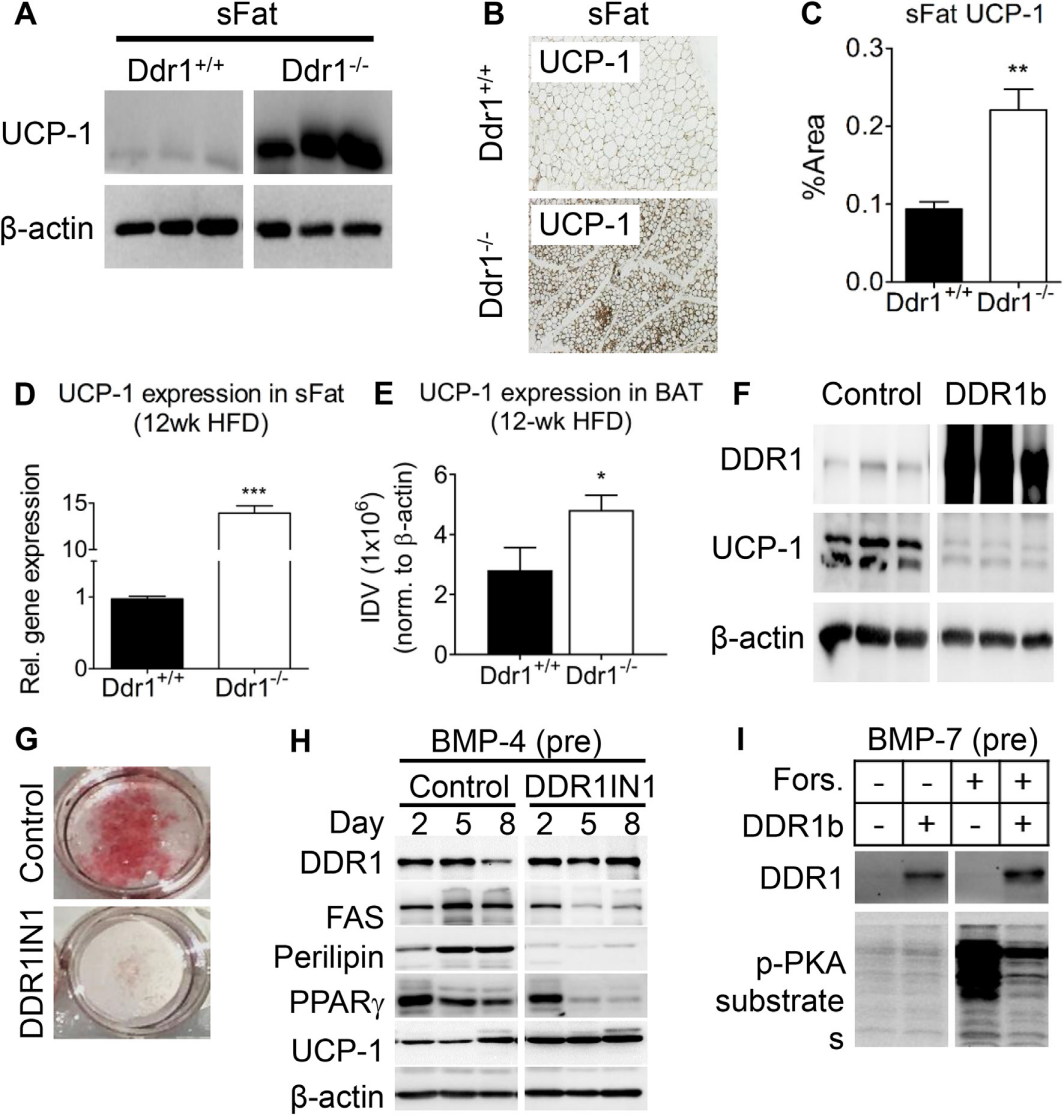
**DDR1 deficient mice have increased UCP-1 in sFat and BAT, while DDR1 overexpression suppressed UCP-1 during adipogenic differentiation *in vitro*.** (**A**) Beige fat marker UCP-1 was assessed by immunoblot in sFat obtained from Ddr1^+/+^ and Ddr1^−/−^ mice after 12 weeks on HFD (n = 6). (**B–C**) Immunostaining was performed to visualize UCP-1 expression (**B**) and quantified (**C**) in sFat adipose tissue of Ddr1^+/+^ and Ddr1^−/−^ mice after 12 weeks on HFD (n = 6). (**D**) UCP-1 mRNA expression in sFat obtained from Ddr1^+/+^ and Ddr1^−/−^ mice after 12 weeks on HFD (n = 4). (**E**) UCP-1 expression was measured in BAT from Ddr1^+/+^ and Ddr1^−/−^ fed HFD (n = 6). (**F**) Undifferentiated C3H10T1/2 cells were transfected with full-length DDR1b isoform or empty vector (control) and probed for UCP-1 expression (n = 3). (**G**) Adipogenic differentiation of C3H10T1/2 cells was induced by treating with BMP-4 and adipogenic cocktail. DDR1 was blocked with the DDR1 inhibitor (DDR1IN1), and lipid droplets were visualized using Oil Red O stain (n = 3). (**H**) During adipogenic differentiation, expression of DDR1, the white adipocyte markers fatty acid synthase (FAS) and perilipin, PPARγ, and the beige fat marker UCP-1 were assessed by immunoblot (n = 3). (**I**) Brown adipogenesis was induced in C3H10T1/2 cells by treating with BMP-7 (6.3 nM), then cells were treated with forskolin to activate cAMP/PKA, and the effect of DDR1 overexpression (DDR1b) on PKA activity was assessed using an antibody against phosphorylated PKA substrate (n = 3). Statistical analysis was performed by Mann-Whitney test (**C**), and student's t-test (**D-E**). The values are mean ± SEM. ∗p < 0.05, ∗∗p < 0.01, ∗∗∗p < 0.001 compared to Ddr1^+/+^.

The appearance of multilocular lipid droplets within subcutaneous adipose prompted us to assess beige fat formation within the white adipose tissue. We assessed the expression of UCP-1, a commonly used marker of beige or brown fat which is not normally found in white adipose tissue. In brown fat, the expression of UCP-1 is induced by cAMP/PKA and results in the uncoupling of cellular respiration resulting in dissipation of energy potential as thermal energy. Immunoblots revealed that UCP-1 was highly expressed in sFat from Ddr1^−/−^ mice, whereas expression was negligible in Ddr1^+/+^ mice (Figure 6A). Immunostaining for UCP-1 was increased 2-fold in sFat from Ddr1^−/−^ compared to Ddr1^+/+^ mice (Figure 6B,C). mRNA for UCP-1 was increased more than 10-fold in the sFat of Ddr1^−/−^ compared to Ddr1^+/+^ mice (Figure 6D). There was also a significant increase in UCP-1 protein level in BAT from Ddr1^−/−^ compared to Ddr1^+/+^ mice fed an HFD (Figure 6E).

The appearance of beige adipose tissue and UCP-1 expression *in vivo* in the DDR1-deficient mice led us to question whether DDR1 affects UCP-1 expression *in vitro* as well as DDR1's potential function in adipocyte differentiation. To investigate this, we used C3H10T1/2 mesenchymal stem cells, which expressed low levels of DDR1. Transfecting the cells to overexpress full-length DDR1b suppressed UCP-1 protein levels (Figure 6F). C3H10T1/2 mesenchymal stem cells were then induced to differentiate into mature adipocytes by stimulating with BMP-4 as previously described [31]. DDR1 inhibition was achieved using DDR1 inhibitor DDR1IN1, which locks DDR1 in the Asp-Phe-Gly (DFG)-out position, thereby blocking autophosphorylation and ligand-mediated activation [33]. Treatment with DDR1IN1 attenuated adipogenesis as evidenced by reduced Oil Red O stain (Figure 6G). DDR1 protein levels did not change during differentiation, nor were they affected by DDR1IN1 treatment (Figure 6H), suggesting that the effects of DDR1 in adipogenesis are mediated via signaling and not changes in receptor levels. Inhibition of signaling with DDR1IN1 resulted in the decreased expression of the white adipocyte markers fatty acid synthase (FAS) and perilipin, and decreased PPARγ (Figure 6H). By contrast, DDR1IN1 increased UCP-1 expression (Figure 6H). These results demonstrate that DDR1 inhibition promotes a shift from white to beige adipogenesis *in vitro*. Because UCP-1 is regulated via cAMP/PKA signaling in brown fat, we determined whether DDR1 could modulate UCP-1 expression via this pathway. Brown adipogenesis was induced in C3H10T1/2 cells by pre-treating with BMP-7 (6.3 nM) and culturing in a specialized brown adipogenic cocktail as previously described [11]. The cells were then treated with forskolin (cAMP pathway activator), which dramatically increased phospho-PKA (Figure 6I). By contrast, the concomitant overexpression of DDR1b blunted forskolin-mediated PKA activation in brown adipocytes (Figure 6I). These results showed that DDR1 overexpression attenuated PKA activity in adipocytes *in vitro*, demonstrating a role for DDR1 in suppressing cAMP/PKA-mediated UCP-1 expression.

### 3.7 DDR1 deficient mice have decreased adipose fibrosis, and DDR1 is upregulated during fibrogenic differentiation of C3H10T1/2 cells *in vitro*

**Figure 7:**
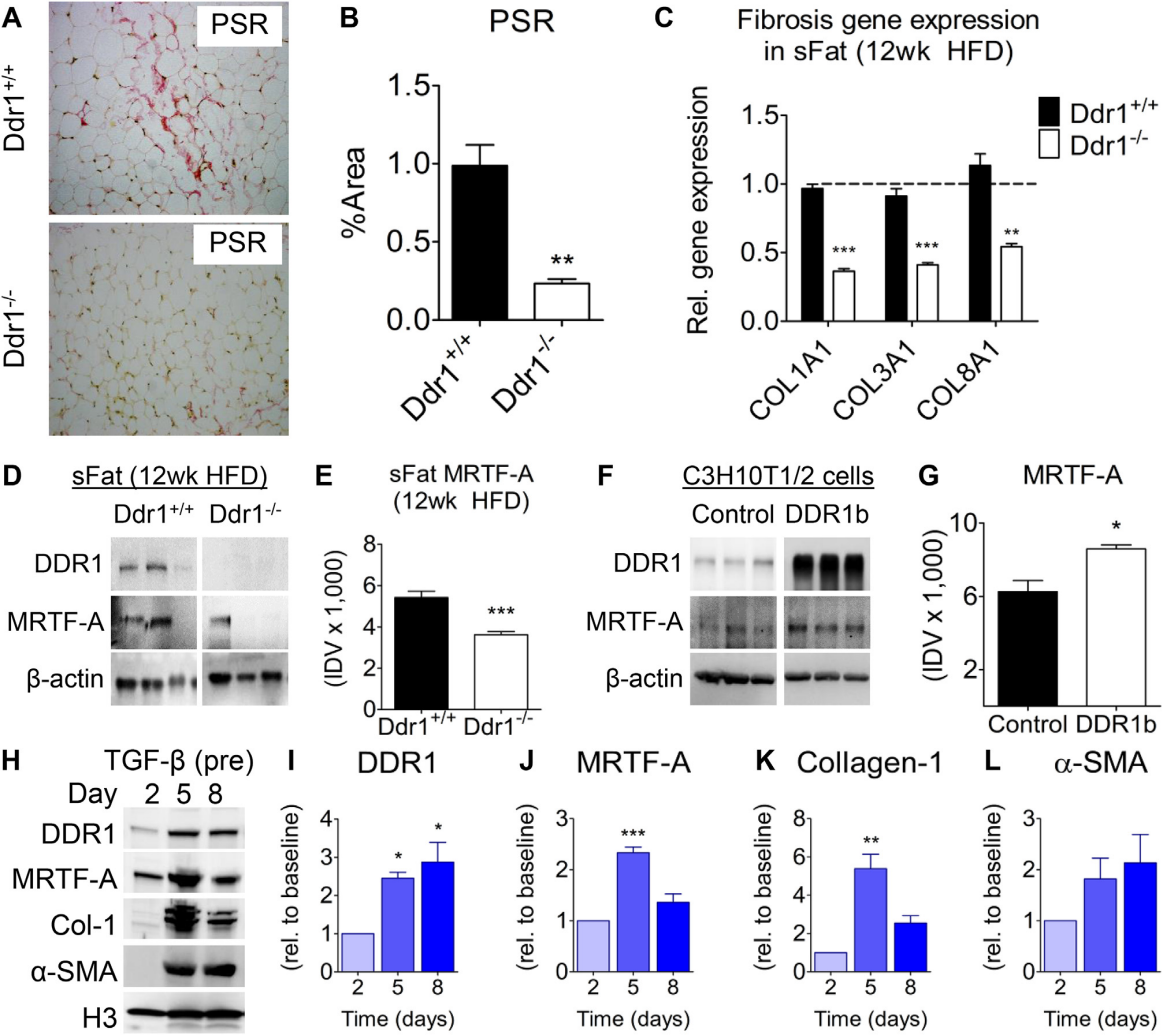
**DDR1 deficient mice have decreased adipose fibrosis, and DDR1 is upregulated during fibrogenic differentiation of C3H10T1/2 cells *in vitro*.** (**A**) sFat from Ddr1^+/+^ and Ddr1^−/−^ mice fed HFD was stained with Picrosirius Red (PSR) to visualize collagen accumulation. (**B**) Peri-adipocyte collagen was quantified (n = 6). (**C**) Collagen mRNA expression in sFat obtained from Ddr1^+/+^ and Ddr1^−/−^ mice after 12 weeks on HFD (n = 4). (**D-E**) DDR1 and MRTF-A protein expression in sFat obtained from Ddr1^+/+^ and Ddr1^−/−^ mice after 12 weeks on HFD (n = 6). (**F-G**) DDR1b was transfected in C3H10T1/2 cells (without differentiation) and MRTF-A expression was assessed by immunoblot (n = 3). (**H-L**) C3H10T1/2 cells were treated with TGFβ to stimulate fibrocyte differentiation, and expression of DDR1 and fibrosis markers was assessed by immunoblot (**H**), and quantified (**I-L**) (n = 3). Statistical analysis was performed using student's t-test (**B**, **C**, **E**, and **G**), and 1-way ANOVA with Bonferroni post-hoc test (**I-L**). The values are mean ± SEM. ∗p < 0.05, ∗∗p < 0.01, ∗∗∗p < 0.001.

Since DDR1 is a potent regulator of tissue fibrosis [16,34,35], and adipose tissue fibrosis is inversely correlated with beiging [12], we reasoned that DDR1 deletion would attenuate fibrosis and allow beiging. We assessed collagen accumulation in sFat by staining with picrosirius red (PSR) dye (Figure 7A). Collagen staining was significantly reduced in Ddr1^−/−^ compared to Ddr1^+/+^ mice after 12 weeks on an HFD (Figure 7B). Furthermore, qRT-PCR measurement of mRNA expression revealed decreased expression of *Col1a1*, *Col3a1*, and *Col8a1* in sFat from Ddr1^−/−^ mice (Figure 7C). This suggests that DDR1 promotes HFD-induced adipose tissue fibrosis.

The myocardin related transcription factor A (MRTF-A) promotes adipose tissue fibrosis by mediating precursor cell differentiation to myofibroblasts [10]. Moreover, previous studies have suggested that MRTF-A suppresses beige adipogenesis because MRTF-A deficient mice have increased beige fat after HFD feeding [11]. To determine whether DDR1 regulates fibrosis via expression of MRTF-A, we probed immunoblots from sFat for MRTF-A and found that levels of MRTF-A were significantly reduced in Ddr1^−/−^ compared to Ddr1^+/+^ mice (Figure 7D,E). Next, we transfected C3H10T1/2 cells to overexpress DDR1b and found that this significantly increased MRTF-A levels (Figure 7F,G). C3H10T1/2 cells were pre-treated with TGFβ as previously described to stimulate differentiation to the fibrotic phenotype [11]. DDR1 expression increased from 2 to 5 days of fibrocyte differentiation (Figure 7I) as did the expression of fibrotic cell markers including MRTF-A (Figure 7J), Collagen I (Figure 7K), though the increase in α-SMA was not significant (Figure 7L). These findings suggest that DDR1 expression is correlated with fibrocyte differentiation and, taken together with the correlation of MRTF-A and fibrosis marker expression, our work suggests that DDR1 mediates tissue fibrosis via an MRTF-A dependent pathway.

## 4 Discussion

The current study was undertaken to evaluate the effect of genetic ablation of the collagen receptor DDR1 in HFD-induced cardiometabolic disease. We demonstrate *in vivo* for the first time a role for DDR1 in the regulation of obesity, glucose tolerance, adipose tissue fibrosis and beiging. Moreover, we present evidence that the mechanism is mediated at least in part through the regulation of adipocyte differentiation and fibrosis. After feeding an HFD for 12 weeks, we observed increased DDR1 expression in white adipose tissue, while conversely, DDR1 deficient mice were leaner despite no changes in food consumption. Additionally, we observed that DDR1 deficient mice had smaller eFat and darker BAT tissue. These findings were reproducible in Ddr1^+/+(BL6)^ and Ddr1^−/−(BL6)^ mice on the C57BL/6N background which were fed the same HFD for 12 weeks, ruling out a confounding effect of LDLR deletion.

Consistent with improved metabolic function, Ddr1^−/−^ mice had improved glucose and insulin tolerance, as well as decreased circulating insulin compared to Ddr1^+/+^ mice. Moreover, pancreatic islet size was increased in Ddr1^+/+^ mice in response to the fat challenge but remained unchanged in Ddr1^−/−^ mice, suggesting that islet expansion occurred primarily in response to the fat challenge and was indirectly mediated by DDR1. To the best of our knowledge, we are the first to report this function for DDR1 in the adult pancreas. Previous studies have reported that DDR1 was expressed during development in pancreatic progenitor cells but not in the adult endocrine pancreas [36], whereas DDR1 was expressed in adult pancreas during tissue regeneration following streptozotocin injury and DPP4 inhibitor treatment [37]. However, neither study investigated functional roles for DDR1. The fact that Ddr1^−/−^ mice develop functional islets suggests that DDR1 is not a critical factor in maintaining the islets. Therefore we turned our attention to DDR1 functions in adipose tissue because maintaining healthy adipose can improve systemic glucose balance, and we observed HFD-induced DDR1 expression in adipose tissue.

Consistent with decreased body weight, CT scanning confirmed a dramatic reduction in body fat in DDR1 deficient mice, showing that the reduction in body weight was not simply due to dwarfing of the DDR1 KO mice, which has been reported previously [38]. Ddr1^−/−^ mice had smaller adipocytes with multi-locular lipid droplets. Expression of mRNA for genes involved in lipid transport, synthesis, and breakdown were also decreased in sFat of HFD-fed Ddr1^−/−^ mice, consistent with the deficiency in lipid content. ^18^F-deoxyglucose uptake was significantly higher in the intrascapular brown fat from DDR1 deficient mice. Similarly, energy expenditure, VO_2_ and VCO_2_ were increased in the DDR1 deficient mice. Although we expected decreased RER, indicating increased lipolytic metabolism, there was no change in RER in Ddr1^−/−^ mice compared to Ddr1^+/+^ mice fed an HFD. This could be due to overall depletion in lipid stores and a switch to metabolizing carbohydrate stores. The fact that we did not see increased activation of HSL in sFat of Ddr1^−/−^ mice is consistent, although it is possible that Ddr1^−/−^ mice have defective dietary lipid absorption, which was not assessed. However, the fact that UCP-1 was increased in sFat and BAT, in addition to increased BAT activity, and energy expenditure, in the absence of changes in locomotor activity, suggest that Ddr1^−/−^ mice are leaner as a consequence of increased energy expenditure.

In the current study, we showed that deficiency of DDR1 *in vivo* led to a dramatic increase in UCP-1 expression in subcutaneous adipose tissue, which suggested browning or beiging of peripheral adipose. Previous research has shown that DDR1 can influence mesenchymal cell differentiation. For example, we have shown that DDR1 mediates osteogenic differentiation of VSMCs during atherosclerotic vascular calcification [23]. Mesenchymal stem cells are multi-potent and capable of differentiating into osteoblasts, adipocytes or fibrocytes, processes that are driven by RUNX2, PPARγ, and MRTF-A, respectively [24,39]. To investigate the mechanisms by which DDR1 influences adipose remodeling, we used C3H10T1/2 mesenchymal stem cells. Adipogenesis was stimulated using an adipogenic cocktail after pretreatment with BMP-4 as previously described [30,31]. Pharmacologic inhibition of DDR1 resulted in reduced differentiation of C3H10T1/2 cells into white adipocytes, as evidenced by reduced expression of white adipocyte markers. By contrast, DDR1 inhibition increased the levels of mRNA and protein for UCP-1, while DDR1 overexpression decreased UCP-1 expression, suggesting that DDR1 loss of function may be causing a shift from white to beige adipogenesis. Furthermore, DDR1 over-expression suppressed forskolin-mediated PKA activation (PKA is the primary mediator of UCP-1 expression), which suggested a cell-autonomous role for DDR1 in the regulation of UCP-1 expression and beige trans-differentiation. This is an important link between the *in vivo* and *in vitro* data.

DDR1 is a collagen binding receptor known to mediate tissue fibrosis by regulating collagen synthesis, deposition and turnover [16,40]. In the current studies, we show *in vivo* that HFD-induced DDR1 expression in subcutaneous fat leads to increased collagen fibrosis, and this was markedly attenuated in DDR1 deficient mice. The transcription factor MRTF-A is a master regulator of genes involved in fibrosis, and we found that MRTF-A levels were dramatically decreased in the sFat of Ddr1^−/−^ mice *in vivo*. This is interesting because recent studies from another group have shown that the differentiation of pro-fibrotic progenitor cells in adipose tissue is controlled by MRTF-A [10,11], and studies show that adipose fibrosis can suppress beige fat formation [12] and lead to adipose dysfunction [4,9,41]. Moreover, MRTF-A deficient mice exhibit reduced obesity, improved glucose tolerance, and increased beige fat and UCP-1 expression [11], a phenotype similar to our DDR1-deficient mice. Therefore we investigated the differentiation of pro-fibrotic progenitors from mesenchymal stem cells *in vitro* and showed that DDR1 controls the expression of MRTF-A and that fibrocyte differentiation was associated with increases in expression of MRTF-A, DDR1, collagen-1 and α-SMA. Though the mechanism is not completely understood, DDR1 promotes RhoA/ROCK activation [17], which could drive MRTF-A and the fibrotic differentiation of C3H10T1/2 cells [11]. Overall, our data support a pro-fibrotic role for DDR1 when it is increased after an HFD leading to adipose dysfunction which suppresses beige fat, whereas DDR1 deletion prevents these changes and allows beige fat to form.

In conclusion, we demonstrate for the first time that DDR1 is upregulated in adipose tissue in a mouse model of cardiometabolic disease. We have uncovered new roles for DDR1 in the regulation of obesity, energy expenditure, adipose tissue fibrosis, and glucose homeostasis *in vivo*, and identified DDR1 as an important regulator of mesenchymal stem cell differentiation *in vitro*. Our findings support a pro-adipogenic and pro-fibrotic role for DDR1 *in vivo*, exacerbating adipose tissue metabolic dysfunction and suppressing beige fat formation. Further research is required to determine the molecular mechanisms by which DDR1 regulates adipose tissue remodeling.

## Author contributions

Conceptualization, M.L. and M.P·B; Methodology, M.L., M.W., and M.P·B; Formal Analysis, M.L.; Investigation, M.L., D.N., A.L., A.M., C·H., S.A.S., and A.G.; Resources, L.L.C., S.A.S., M.W., and M.B·P.; Writing-Original Draft, M.L.; Writing-Review & Editing, M.L., M.P·B., A.G. and M.W.; Supervision, M.P·B., M.W. and A.G. Funding Acquisition, M.L., M.P·B., and A.G.
