# Supplementary material for: Discoidin domain receptor 1-deletion ameliorates fibrosis and promotes adipose tissue beiging, brown fat activity, and increased metabolic rate in a mouse model of cardiometabolic disease
Source: Mol Metab. 2020 Apr 28;39:101006. doi: 10.1016/j.molmet.2020.101006 (PMC7242876; doi:10.1016/j.molmet.2020.101006)
Supplement: Multimedia component 1 [file mmc1.pdf]

**Table S1. Primers sequences for genes assessed by qRT-PCR.**

| Gene | Forward primer (5' to 3') | Reverse primer (5' to 3') |
|---|---|---|
| *ucp1* | CTGCCTCTCTCGGAAACAAG | TGCATTCTGACCTTCACGAC |
| *col1a1* | ACGCCATCAAGGTCTACTGC | GTTCGGGCTGATGTACCAGT |
| *col3a1* | ACCAAAAGGTGATGCTGGAC | TCCAGTTAGCCCTGCAATTC |
| *col8a1* | GGGATTTTGGAGGGTTCTTC | TAGTCCTGCAGCTCAAAGCA |
| *srebp2* | AGCTGGCAAATCAGAAAAACAAG | GATTAAAGTCTTCAATCTTCAAGTCCAC |
| *cd36* | GTATTCTCATGCCAGTTGGAGAC | TTTAACCCAGTTTTTGAAAGCAA |
| *abcg1* | AAGAAGGTGGACAACAACTTCAC | CCTTTCAAAAGGGTCTTGTATCC |
| *abcg5* | CCATTCTGACTTACGGAGAGTTG | CAGGGGTAACCACAGTTATTGAA |
| *fas* | GCAGTCTTGAGTAGCTTTGTGCT | GGGAGCTGTCCAGATTAATACCT |
| *lpl* | TACAGGTGCAACTCTAAGGAAGC | GTCTTCAGGTACATCTTGCTGCT |
| *18s* | CGAAAGCATTTGCCAAGAAT | AGTCGGCATCGTTTATGGTC |

Primers for *ucp1*, *col1a1*, *col3a1*, *col8a1*, and *18s* were generated using Batch Primer 3 v1.0 (http://batchprimer3.bioinformatics.ucdavis.edu/cgi-bin/batchprimer3/batchprimer3.cgi). Primers for *srebp2*, *cd36*, *abcg1*, *abcg5*, *fas*, and *lpl*, were published elsewhere [1].

1. Naples, M., C. Baker, M. Lino, J. Iqbal, M.M. Hussain and K. Adeli, 2012. Ezetimibe ameliorates intestinal chylomicron overproduction and improves glucose tolerance in a diet-induced hamster model of insulin resistance. Am J Physiol Gastrointest Liver Physiol 302(9): G1043-52. https://doi.org/10.1152/ajpgi.00250.2011.

**Table S2. Fasting blood glucose, triglyceride, total cholesterol, HDL-c and LDL-c in $Ddr1^{+/+}$ and $Dd1^{-/-}$ mice fed HFD or control CF for 12 weeks.**

| Parameter | $Ddr1^{+/+}$ (n=9) | $Ddr1^{-/-}$ (n=9) | p-value | Sig |
|---|---|---|---|---|
| CF Body weight (g) | 21.90±2.34 | 18.53±4.28 | 0.0316 | * |
| HFD Body weight (g) | 43.10±5.33 | 31.65±6.87 | 0.0008 | *** |
| CF Blood glucose (mg/dL) | 137.90±25.96 | 121.70±43.34 | 0.3009 | ns |
| HFD Blood glucose (mg/dL) | 167.80±24.76 | 152.20±22.09 | 0.1772 | ns |
| HFD Triglyceride (mg/dL) | 214.80±23.31 | 189.90±26.43 | 0.4946 | ns |
| HFD Total Cholesterol (mg/dL) | 1694±106.2 | 1031±104.6 | 0.001 | *** |
| HFD HDL-c (mg/dL) | 101.70±3.25 | 88.52±7.93 | 0.1149 | ns |
| HFD LDL-c (mg/dL) | 374.90±41.02 | 229.80±15.11 | 0.0127 | * |

CF, 12-week chow-fed; HFD, 12-week high-fat diet fed; HDL-c, high-density lipoprotein cholesterol; LDL-c, low-density lipoprotein cholesterol. Some of these data were previously published [1].

1. Lino, M., M.H. Wan, A.S. Rocca, D. Ngai, N. Shobeiri, G. Hou, et al., 2018. Diabetic Vascular Calcification Mediated by the Collagen Receptor Discoidin Domain Receptor 1 via the Phosphoinositide 3-Kinase/Akt/Runt-Related Transcription Factor 2 Signaling Axis. Arterioscler Thromb Vasc Biol 38(8): 1878-1889. https://doi.org/10.1161/ATVBAHA.118.311238.

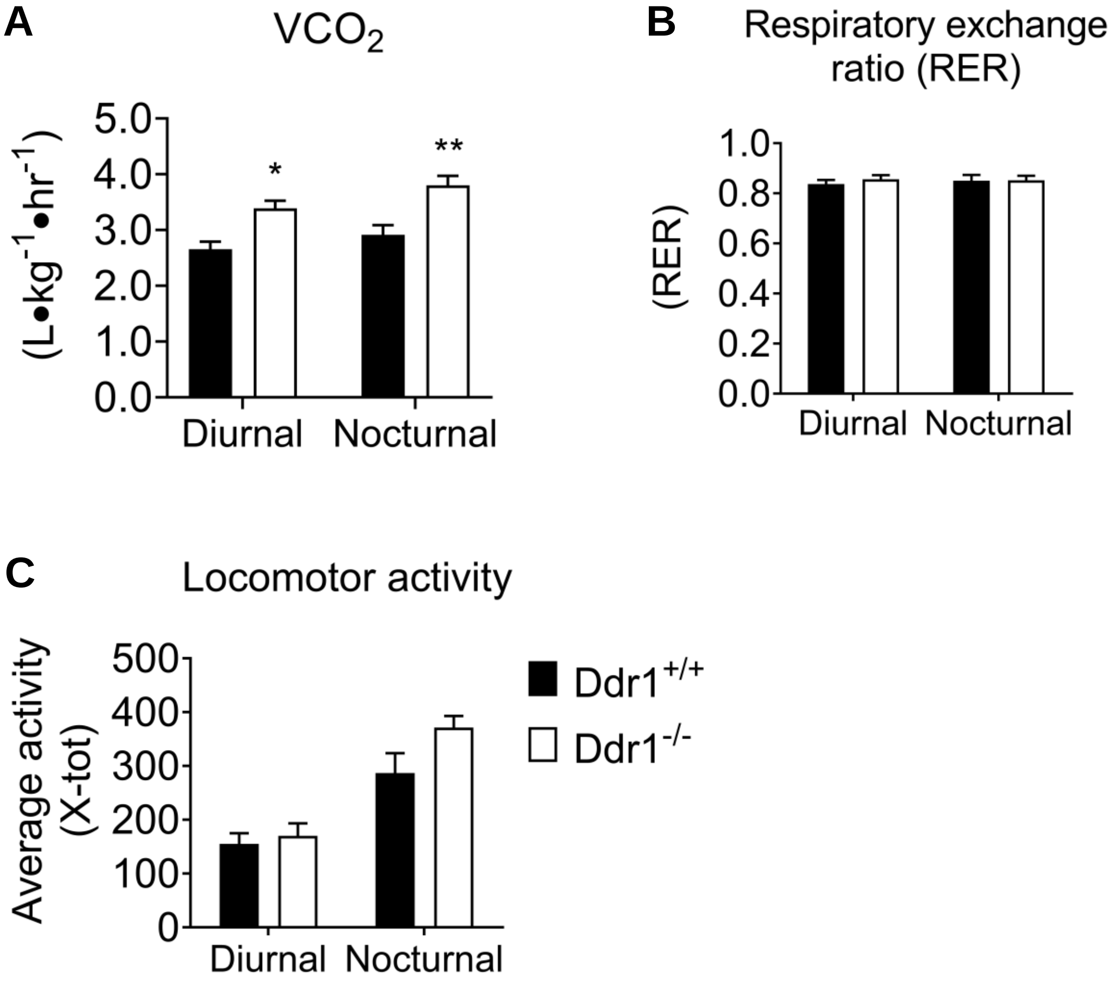


**Figure S1. $Ddr1^{-/-}$ mice have increased $VCO_2$, no change in RER or locomotor activity.** (**A-C**) $VCO_2$ (**A**), respiratory exchange ratio (RER) (**B**), and locomotor activity (**C**), were measured in $Ddr1^{+/+}$ and $Ddr1^{-/-}$ mice fed HFD for 6 weeks. Statistical analysis was performed by 2-way ANOVA with Bonferroni post-hoc test (**A-C**). $^*p<0.05$, $^{**}p<0.01$, compared to $Ddr1^{+/+}$.
